# Extracts from the Records of the Suffolk District Medical Society

**Published:** 1858-04

**Authors:** Charles D. Homans

**Affiliations:** Secretary


*Extracts from the Records of the Suffolk District Medical Society. Charles D. Homans, M. D., Secretary.*

[*Imperforate Rectum.**—A male child, 3 days old, had passed nothing from its bowels since its birth. Its abdomen was distended, the skin was yellow, and there was some vomiting.

The anus was normal; the little finger could be introduced to the distance of about three-fourths of an inch, when it encountered an obstacle which prevented its further passage.

*Operation.*—An exploring needle was passed in by Dr. H. G. Clark, the consulting surgeon, to the distance of an inch above the termination of the cul de sac. This was followed by the discharge of gas through the canula. The opening was immediately dilated by bougies of different sizes up to that of a common catheter, and meconium was freely discharged. The dilatation was followed up by Dr. Moore, and the child was well in one week.]

* This case of Dr. Moore having been inaccurately reported on page 510, of our last volume, is her reprinted in a corrected form.

June 27th, 1857.—Dr. Buck reported two cases of *Erysipelas following Vaccination.* They were novel to him, and might be interesting to the Society.

In March last, he vaccinated a child a few weeks old, and at the end of a week a good vesicle had formed; there was no areola. He charged some quills, and supposed that everything would go on according to the rule. The third day after this, the child was attacked with erysipelas about the pustule, which soon involved the whole arm, and extended to the chest, running its course very rapidly. Death ensued, on the eighth day from the time when the disease was first seen. He vaccinated several children with matter taken from this child, and they all did well.

On the 4th of the present month, he vaccinated a healthy child, also a few weeks old, and on the seventh day charged his quills. Nine days after this, the sixteenth day after the vaccination, erysipelas appeared on the arm, ran down the chest, abdomen and the legs; this patient is now recovering. He also vaccinated several children with lymph taken from this case, and no trouble had as yet ensued.

There was no case of erysipelas in the houses where either of these children were living, and no predisposition to this disease, as far as he knew, in either of the families to which they belonged. These are the only cases of the kind Dr. Buck has ever met with.

Dr. Hodges referred to the report of two cases of *Hydrocele treated by the red oxide of mercury,* printed in the Extracts from the Records of this Society, in the last number of the Medical Journal. This mode of treatment was first recommended by Mr. Lake, of London. Since those two cases were reported, Dr. Hodges had had occasion to try this method. After the fluid had been withdrawn, about five grains of the red oxide were pushed through the canula by means of a probe. The inflammation which followed was not greater than that generally produced by the injection of iodine, but salivation ensued on the fifth or sixth day. The hydrocele was cured. This accident had not been mentioned in the reports of any of the case which Dr. Hodges had seen. The red oxide of mercury was formerly a favorite application to ulcers, and was not generally followed by salivation.

Dr. Buck stated that, thirty years since, he had been in the habit of frequently using this preparation of mercury as an application to ulcers, but he had never seen salivation as a result of it.

Dr. Hodges thought the large absorbing surface of the tunica vaginalis a sufficient explanation of its occurrence in the case on which he had operated. He considered it an important fact to remember, as it had not been mentioned, so far as he knew, as an accideut likely to occur.

Dr. Buck asked what advantage there was in the use of this drug over the tincture of iodine.

Dr. Hodges considered its greater convenience its ouly recommendation. A surgeon could carry it about with him much more easily than a bottle of the tincture of iodine and a syringe. He thought, however, that he should not try it again himself.

Dr. M. C. GREENE reported a case of *fungous growth from the navel.* The cord had sloughed off from an infant a week after birth, leaving a point of ulceration about the size of a pin's head. This immediately began to increase, and Dr. Greene was called in ten days after its first appearance.—It was then about six lines in diameter, and eight in height. Astringents and caustics seemed to have no effect upon it. A ligature was applied, and in two days all was well. There was occasionally hæmorrhage. He had never seen a case of the kind before.

Dr. C. G. PAGE had meet with an instance of the same affection, which, after a similar treatment, was followed by recovery· In this case, also, caustic and astringent applications seemed to be of no benefit.

*Blighted Ovum.*—Dr. A. B. HALL reported the case and exhibited the specimen. The patient from whom it came away considered herself as in the fourth month of pregnancy. She had been confined eighteen months previously. Saturday evening, she was taken in labor, and a hydatid cyst was soon thrown off, which would countain perhaps two ounces of fluid. On Sunday morning, six hours later, the mass on the table was thrown off, and the labor was finished. The specimen was about the size of a cocoanut, consisting apparently of the fœtal membranes. It was an empty sac, the walls at one bar being much thickened; their structure at that place resembling that of the placenta. A rupture existed nearly opposite this portion, through which Dr. Hall supposed the hydatid had passed.—*Boston. Med. & Sur. Journal.*

